# Supplementary material for: A circuit mechanism for decision-making biases and NMDA receptor hypofunction
Source: eLife. 2020 Sep 29;9:e53664. doi: 10.7554/eLife.53664 (PMC7524553; doi:10.7554/eLife.53664)
Supplement: Supplementary file 1. — Log-likelihood values were calculated using a cross-validation procedure (see Materials and methods). Column label refers to the removed regressor. Positive values indicate the full regression model performs better. Values depend on the number of completed trials, which differed both between subjects and the circuit model. For both monkeys and the circuit model, mean evidence is clearly the most important driver of choice behaviour, followed by first and last evidence samples which reflects the primacy bias. Finally, evidence standard deviation (SD) has a stronger effect than maximum and minimum evidence samples (Max and Min). [file elife-53664-supp1.docx]

|  | Mean | First & Last | SD | Max & Min |
| --- | --- | --- | --- | --- |
| Monkey A | 990 | 66.8 | 15.1 | -1.33 |
| Monkey H | 1270 | 61.0 | 6.82 | 5.38 |
| Circuit Model | 6170 | 1860 | 96.9 | 42.5 |

***Supplementary File 1:*** *Difference in log-likelihood of Full regression model (mean, SD, max, min, first, last of evidence values; equation 6 in* ***Methods****) vs reduced model, for each monkey and the circuit model. Log-likelihood values were calculated using a cross-validation procedure (see* ***Methods****). Column label refers to the removed regressor. Positive values indicate the full regression model performs better. Values depend on the number of completed trials, which differed both between subjects and the circuit model. For both monkeys and the circuit model, mean evidence is clearly the most important driver of choice behaviour, followed by first and last evidence samples which reflects the primacy bias. Finally, evidence standard deviation (SD) has a stronger effect than maximum and minimum evidence samples (Max & Min).*
