# Supplementary material for: A circuit mechanism for decision-making biases and NMDA receptor hypofunction
Source: eLife. 2020 Sep 29;9:e53664. doi: 10.7554/eLife.53664 (PMC7524553; doi:10.7554/eLife.53664)
Supplement: Supplementary file 2. — Log-likelihood values were calculated using a cross-validation procedure (see Materials and methods). Column label refers to the regressors additional to either SD or Max and Min. Positive values indicate the regression model with SD performs better than that with Max and Min. Values depend on the number of completed trials, which differed both between subjects and the circuit model. Regardless of whether first and last evidence sample regressors are included, the models with standard deviation of evidence have higher log-likelihoods than the models with maximum and minimum evidence samples, indicating a better explanation of the data by standard deviation than by maximum and minimum evidence samples. [file elife-53664-supp2.docx]

|  | Mean | Mean & First & Last |
| --- | --- | --- |
| Monkey A | 15.2 | 16.4 |
| Monkey H | 1.81 | 1.44 |
| Circuit Model | 54.5 | 56.7 |

***Supplementary File 2:*** *Difference in log-likelihood of regression models including either evidence standard deviation (SD) or both maximum and minimum evidence (Max & Min) as regressors, for each monkey and the circuit model. Log-likelihood values were calculated using a cross-validation procedure (see* ***Methods****). Column label refers to the regressors additional to either SD or Max & Min. Positive values indicate the regression model with SD performs better than that with Max & Min. Values depend on the number of completed trials, which differed both between subjects and the circuit model. Regardless of whether first and last evidence sample regressors are included, the models with standard deviation of evidence have higher log-likelihoods than the models with maximum and minimum evidence samples, indicating a better explanation of the data by standard deviation than by maximum and minimum evidence samples.*
