# Supplementary material for: A circuit mechanism for decision-making biases and NMDA receptor hypofunction
Source: eLife. 2020 Sep 29;9:e53664. doi: 10.7554/eLife.53664 (PMC7524553; doi:10.7554/eLife.53664)
Supplement: Supplementary file 3. — Log-likelihood values were calculated using a cross-validation procedure (see Materials and methods). Values depend on the number of completed trials, which differed both between subjects and the circuit model. Positive values across the table indicates the evidence standard deviation regressor robustly improves model performance for all models examined. [file elife-53664-supp3.docx]

|  | Mean | Mean, First, & Last | Mean, Max, & Min | Mean, Max, Min, First, & Last |
| --- | --- | --- | --- | --- |
| Monkey A | 189 | 200 | 15.3 | 15.1 |
| Monkey H | 74.8 | 75.5 | 5.84 | 6.82 |
| Circuit Model | 1000 | 1070 | 97.1 | 96.9 |

***Supplementary File 3:*** *Increase in log-likelihood of various regression models (regressors in column labels) due to inclusion of evidence standard deviation as a regressor, for each monkey and the circuit model. Log-likelihood values were calculated using a cross-validation procedure (see* ***Methods****). Values depend on the number of completed trials, which differed both between subjects and the circuit model. Positive values across the table indicates the evidence standard deviation regressor robustly improves model performance for all models examined.*
