# Supplementary material for: A circuit mechanism for decision-making biases and NMDA receptor hypofunction
Source: eLife. 2020 Sep 29;9:e53664. doi: 10.7554/eLife.53664 (PMC7524553; doi:10.7554/eLife.53664)
Supplement: Transparent reporting form [file elife-53664-transrepform.docx]

***eLife’s* transparent reporting form**

We encourage authors to provide detailed information *within their submission* to facilitate the interpretation and replication of experiments. Authors can upload supporting documentation to indicate the use of appropriate reporting guidelines for health-related research (see [EQUATOR Network](http://www.equator-network.org/%20)), life science research (see the [BioSharing Information Resource](https://biosharing.org/)), or the [ARRIVE guidelines](http://www.plosbiology.org/article/info:doi/10.1371/journal.pbio.1000412) for reporting work involving animal research. Where applicable, authors should refer to any relevant reporting standards documents in this form.

If you have any questions, please consult our Journal Policies and/or contact us: [editorial@elifesciences.org](mailto:editorial@elifesciences.org).

**Sample-size estimation**

- You should state whether an appropriate sample size was computed when the study was being designed
- You should state the statistical method of sample size computation and any required assumptions
- If no explicit power analysis was used, you should describe how you decided what sample (replicate) size (number) to use

Please outline where this information can be found within the submission (e.g., sections or figure legends), or explain why this information doesn’t apply to your submission:

Sample size for most analyses was the number of trials performed.

This information can be found:

- Standard Experimental Sessions: Figures 2-4

Second paragraph of results section:

*“Two monkeys (Macaca mulatta) completed 29,726 trials (Monkey A: 10,748; Monkey H: 18,978).”*

- Spiking circuit model (control): Figure 5

Spiking circuit model subsection of methods section:

*“The control circuit model completed 94,000 ‘Regular’ trials, where both streams were narrow in 25% of the trials, both streams were broad in 25% of the trials, and one stream was narrow and one was broad in 50% of the trials (****Figure 5, Figure 5-figure supplement 1,2****). All trials were generated identically as in standard session experiments. The control model also completed 47,000 standard session Narrow-Broad trials.*

- Mean Field model: Figure 6

Mean field model subsection of methods section:

*“The mean-field model completed 94,000 standard session ‘Regular’ trials, in the same manner as the circuit models.”*

- Spiking circuit model (perturbation): Figure 7 and related supplements

Spiking circuit model subsection of methods section:

“*To evaluate the effect of circuit perturbations, the control model, the lowered E/I model, the elevated E/I model, and the sensory deficit model all completed an identical set of 40,000 ‘Regular’ trials, where both streams were narrow in 25% of the trials, both streams were broad in 25% of the trials, and one stream was narrow and one was broad in 50% of the trials (****Figure 7, Figure 7-figure supplement 1****).*

- Pharmacological Experimental Sessions: Figure 8

Near the end of the results section:

*“The data collected during this period formed a behavioural database of 8521 completed trials (Monkey A Saline: 1710; Monkey A Ketamine: 2276; Monkey H Saline: 2669; Monkey H Ketamine: 1866).”*

No explicit power analysis was used for these sample sizes. The number of experimental sessions (and hence trials) was according to common standards in the field.

**Replicates**

- You should report how often each experiment was performed
- You should include a definition of biological versus technical replication
- The data obtained should be provided and sufficient information should be provided to indicate the number of independent biological and/or technical replicates
- If you encountered any outliers, you should describe how these were handled
- Criteria for exclusion/inclusion of data should be clearly stated
- High-throughput sequence data should be uploaded before submission, with a private link for reviewers provided (these are available from both GEO and ArrayExpress)

Please outline where this information can be found within the submission (e.g., sections or figure legends), or explain why this information doesn’t apply to your submission:

Number of experiments (and replications): Methods section.

*“Subjects performed two types of behavioural sessions: standard and pharmacological. In pharmacological sessions, following a baseline period, either an NMDA-R antagonist (Ketamine) or saline was administered via intramuscular injection. Monkey A completed 41 standard sessions, and 28 pharmacological sessions (15 ketamine; 13 saline). Monkey H completed 68 standard sessions, and 35 pharmacological sessions (18 ketamine; 17 saline).”*

Biological Replications:

It is widely accepted in non-human primate neuroscience studies to use only two subjects. Importantly, we also confirmed the same effects were present in both monkeys – each figure separated by subjects is included as supplementary material.

No outliers were encountered.

The only case of excluded data was in the pharmacological experiment. We only excluded data when insufficient trials were performed in a ketamine session. This is reported in the methods section:

*“Monkey H did not always complete sufficient trials once ketamine was administered. Sessions where the number of completed trials was fewer than the minimum recorded in the saline sessions were discarded (6 of 18 sessions). Following ketamine administration, Monkey A did not complete fewer trials in any session than the minimum recorded in a saline session.”*

**Statistical reporting**

- Statistical analysis methods should be described and justified
- Raw data should be presented in figures whenever informative to do so (typically when N per group is less than 10)
- For each experiment, you should identify the statistical tests used, exact values of N, definitions of center, methods of multiple test correction, and dispersion and precision measures (e.g., mean, median, SD, SEM, confidence intervals; and, for the major substantive results, a measure of effect size (e.g., Pearson's r, Cohen's d)
- Report exact p-values wherever possible alongside the summary statistics and 95% confidence intervals. These should be reported for all key questions and not only when the p-value is less than 0.05.

Please outline where this information can be found within the submission (e.g., sections or figure legends), or explain why this information doesn’t apply to your submission:

This information can be found in the Methods section, results section, and figure legends.

(For large datasets, or papers with a very large number of statistical tests, you may upload a single table file with tests, Ns, etc., with reference to sections in the manuscript.)

**Group allocation**

- Indicate how samples were allocated into experimental groups (in the case of clinical studies, please specify allocation to treatment method); if randomization was used, please also state if restricted randomization was applied
- Indicate if masking was used during group allocation, data collection and/or data analysis

Please outline where this information can be found within the submission (e.g., sections or figure legends), or explain why this information doesn’t apply to your submission:

We randomly allocated experimental conditions on a trial-by-trial basis (e.g., mean bar height; standard deviation of bar height). This information was reported in the Methods section.

**Additional data files (“source data”)**

- We encourage you to upload relevant additional data files, such as numerical data that are represented as a graph in a figure, or as a summary table
- Where provided, these should be in the most useful format, and they can be uploaded as “Source data” files linked to a main figure or table
- Include model definition files including the full list of parameters used
- Include code used for data analysis (e.g., R, MatLab)
- Avoid stating that data files are “available upon request”

Please indicate the figures or tables for which source data files have been provided:

Data and analysis scripts to reproduce figures from the paper will be made publicly available for download from an online repository upon publication.
